# Cortical Contrast Processing in Retinitis Pigmentosa: Evidence of PVEPs Spatial Functions

**DOI:** 10.3390/ejihpe10040071

**Published:** 2020-10-16

**Authors:** Rafael Mancebo-Azor, José Antonio Sáez-Moreno, José Manuel Rodríguez-Ferrer

**Affiliations:** 1 Laboratory of Visual and Cognitive Neuroscience, Institute of Neuroscience, University of Granada, 18016 Granada, Spain; manazor@correo.ugr.es (R.M.-A.); josea.saez.sspa@juntadeandalucia.es (J.A.S.-M.); 2 Department of Clinical Neurophysiology, San Cecilio University Hospital, 18016 Granada, Spain; 3 Department of Physiology, University of Granada, 18016 Granada, Spain

**Keywords:** pattern reversal visual evoked potentials, P100-spatial functions, retinitis pigmentosa, visual cortex

## Abstract

Objective: To study the effect of check width size of the stimuli on the amplitude and latency of the P100 component of visual evoked potentials recorded in patients with retinitis pigmentosa (RP). Methods: Pattern reversal visual evoked potentials (PVEPs) were recorded in 16 RP patients and 20 visually normal subjects. Pattern reversal stimuli with five different check widths and 100% of contrast were projected in the right eye of both patients and control subjects. PVEPs induced by stimuli with 78%, 16%, and 6% of contrast were also recorded in 10 of the control subjects. Results: In RP patients, the amplitude of P100 was smaller than controls in all check sized used and the peak P100 amplitude was obtained with a larger check width than in controls. P100 was also delayed in RP patients in all check sizes studied. The P100 amplitude- and latency-check size functions of RP patients were like those found in control subjects with low contrast stimuli of 16% and 6%. Conclusion: The PVEPs spatial functions of RP patients show quantitative and qualitative changes, suggesting disease induced alteration in the neural processing of stimulus contrast.

## 1. Introduction

Retinitis pigmentosa (RP) refers to a heterogeneous group of inherited, progressive, retinal disorders characterized by abnormalities of the photoreceptors and the retinal pigment epithelium that lead to progressive visual loss [1,2]. RP has a combined incidence of approximately 1/3500. In typical cases, known as rod-cone RP, the rods are the predominantly affected cells, and the main symptoms are night blindness and bilateral symmetric loss of peripheral visual field. Although there is usually relative preservation of macular vision, with progression, cone photoreceptor cells are also affected, and day vision and visual acuity are compromised. The variant that involves simultaneous cone photoreceptor degeneration, referred as cone-rod RP, shows a more central loss of the visual field and greater early changes in cone dominated central retina. RP is associated with an expanding number of newly identified gene mutations that alter the visual transduction cascade and photoreceptor transcription and structure [3,4,5]. At present, there is no therapy for stopping the progression of the disease. However, vision rescue strategies are expected in the development of protective neurotrophic agents, gene therapy, retinal transplantation, and bionic implants of stem/engineer cells [6,7,8,9].

There are several ways of monitoring the visual loss in patients with RP. Objective electrophysiological tools, such as the visual evoked potentials (VEP), have been of major importance for this purpose [10]. The VEP, resulting from signal averaging of the electroencephalographic activity recorded at the occipital pole, provide information regarding the functional integrity of the visual system, from the retina to the visual cortex [11]. Pattern reversal visual evoked potentials (PVEPs), which use as stimuli black and white checks that change phase abruptly, is the preferred method since the results are less variable in waveform and timing than those elicited by other stimuli [12]. PVEPs generated in the occipital cortex represent the primary cortical visual processing elicited by stimulation of the ganglion cells in the macula. PVEPs recordings consist of N75, P100, and N135 peaks, designating peaks as negative and positive followed by the typical peak latency in ms. The P100 peak is the most robust PVEPs component and valuable for clinical diagnosis since it shows relatively little variation between normal subjects, minimal within-subject interocular difference, and minimal variation with repeated measurements over time [11,12].

In RP patients, recording of PVEPs is particularly useful to objectively evaluate the residual visual capacity since the activity of the visual cortex can be detected in those patients with no response to other electrophysiological techniques, such as the electroretinogram [13]. In previous studies, PVEPs recordings in RP using a single check width [13,14] showed an attenuation of amplitude of the P100 and, in some cases, delayed latency, but the effects of different check widths P100 amplitude and latency-spatial functions have not yet been described in these patients. Since the amplitude and latency of P100 are sensitive to the check size of the stimuli [15,16], the aim of the present study was to investigate in RP patients the effects of different check widths in both the amplitude and latency of P100. Using different check widths will also allow us to obtain P100 amplitude- and latency-check size functions of RP patients.

## 2. Materials and Methods

A total of 16 patients with RP (age range 23–57 years, 4 males) participated in the study. The clinical characteristics of the patients are listed in Table 1. The inclusion criteria were the ophthalmic appearance of typical RP, with narrow retinal arteries, bone spicule pigmentation, or diffuse retinal pigment epithelium degeneration, and an abnormal or non-recordable electroretinogram. Patients were excluded if they had ophthalmic disorders (e.g., cataract) or neuroophthalmic symptoms (e.g., nystagmus), or if they were under any kind of topical or systemic medication. All RP patients included in the study had night blindness, constriction of the visual field and, except for two patients, reduction of visual acuity. The normal controls were 20 subjects (age range 19–60 years, 8 males). They were healthy, on no medication, had no history of ophthalmic or neurological disorders, and their visual acuity was 1.0 or better. The study adhered to the tenets of the Declaration of Helsinki, and it was approved by the Ethical Committee of the University of Granada. An informed written consent was obtained from all subjects.

The recording of PVEPs followed the standard of the International Society for Clinical Electrophysiology of Vision [11]. Subjects were positioned 114.5 cm from the stimulus display. The PVEPs were recorded monocularly (right eye) in a dark room. The active electrode was positioned at the Oz (International 10–20 system), the reference electrode at Fz and ground electrode at Cz. Electrode impedance was below 1 kΩ. We used the square-wave, checkerboard, pattern reversal stimulus (1 Hz) presented by a television monitor (EEVOKE system, from Advance Neurotechnology software BV, ANT systems. Enschede, The Netherlands). The overall stimulus field was 24 cm × 32 cm, subtending a visual angle of 11.8° × 15.6°. We measured the PVEPs using five different sizes of black and white checks: 60′, 30′, 15′, 7.5′, and 3.75′ (minutes of arc) of check width, corresponding to spatial dominant frequencies of 0.71, 1.41, 2.83, 5.66, and 11.31 cycles per degree (cpd), respectively. The Michelson contrast({[Lmax − Lmin]/[Lmax + Lmin]} × 100) was 100%, where L = luminance, max = maximum and min = minimum, and the mean luminance was 50 Cd/m2. To study the effects of stimulus contrast on PVEPs, in 10 of the control subjects, stimuli of 78%, 16%, and 6% of contrast was also used. The PVEPs elicited were fed into a signal-averaging computer system (Advance Source Analysis, ANT systems. Enschede, The Netherlands). The signals were amplified and passed through a band-pass filter at between 1 Hz and 100 Hz. Experimental sessions consisted of the presentation of 200 stimuli, and the analysis time was 500 ms.A mean of 195 stimuli were averaged for analysis. The results were analyzed by a one-way ANOVA and Bonferroni tests with STATA 10.1 software.Data were presented as the mean ±SEM. *p* < 0.05 was considered statistically significant.

## 3. Results

**Figure 1:**
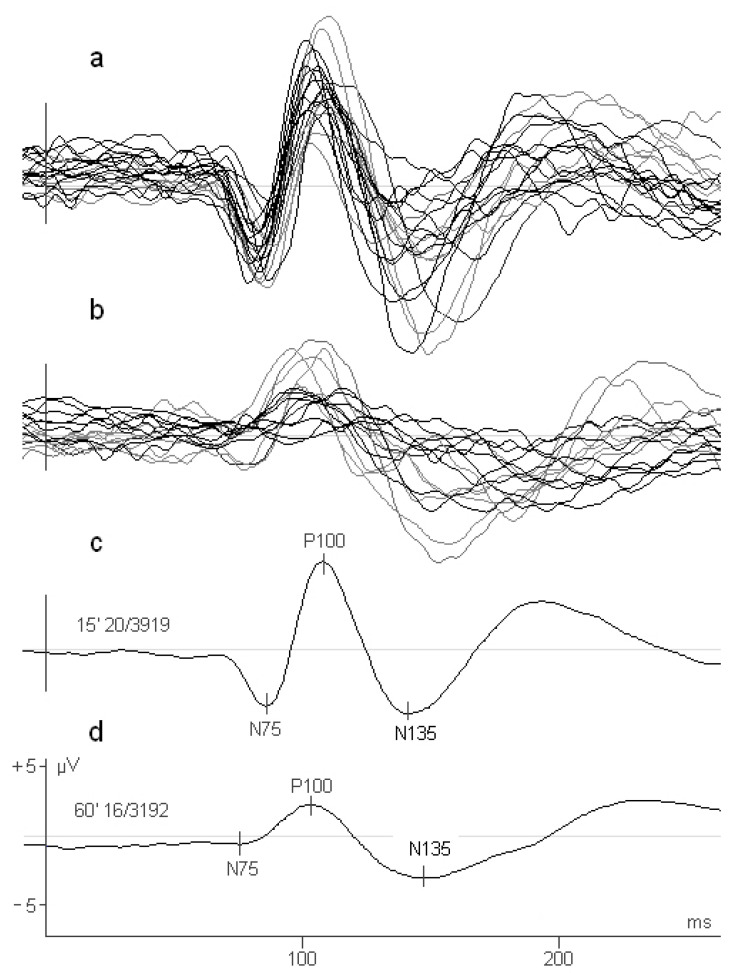
PVEPs obtained in (**a**) control subjects and (**b**) RP patients with the check width that produced the largest amplitude of the P100; 15′ and 60′, respectively. (**c**,**d**) averaged PVEPs of controls and patients, respectively. N75, P100 and N135 peak components are indicated.

Figure 1 shows individual PVEPs recording obtained in 100% of contrast and with the check width where controls and RP patients had the largest amplitude (15′ and 60′, respectively). The mean amplitude of P100 (measured from N75 peak) was 10.8 µV for controls and 2.71 µV for RP patients. In contrast to controls, differences in the amplitude of P100 in RP patients (Figure 1b) clearly show the existence of two groups of patients, one of each with larger amplitude of the PVEPs.

The first group called RP1 was composed of patients 1–8, and the second called R2 was composed of patients 9–16 (Table 1). RP1 group had better visual acuity and larger amplitude of PVEPs than the second group of patients (Table 2). Thus, RP1 group shows a mean visual acuity of 0.62 in decimal scale, peak P100 amplitude between 4.12 µV and 9.7 µV, and mean of age 37.9 ± 12.22 years. RP2 group had a mean visual acuity of 0.31, peak P100 amplitude between 1.25 µV and 4 µV, and mean of age 45.5 ± 9.17 years. Differences in visual acuity and P100 amplitude were significant between both groups (*p* < 0.01), but differences in age were not. To have a control group equivalent in age with RP patients, healthy subjects were divided in two groups of 10 individuals (Table 2), C1 (mean of age 19.3 ± 0.48 years, 4 males) and C2 (mean of age 37.5 ± 10.74 years, 4 males). In RP1, RP2, and C2 groups the contrast stimuli used was 100%. In C1 group, stimuli were presented with contrasts of 100%, 78%, 16%, and 6%. Table 2 shows the P100 amplitude and latency obtained with the different contrasts and in the four experimental groups.

**Figure 2:**
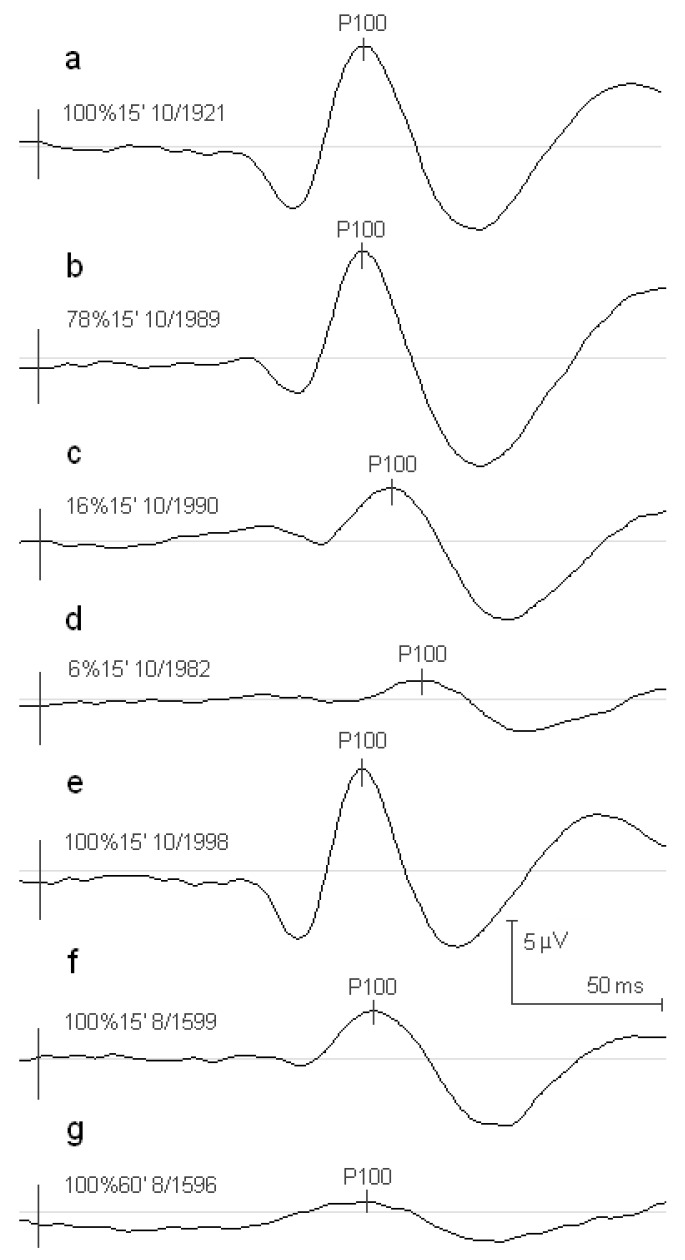
(**a**–**d**) Averaged PVEPs obtained in the C1 control group with the check size of 15′ and contrasts of 100%, 78%, 16%, and 6%, respectively. (**e**) Averaged PVEPs obtained in the C2 control group with the check size of 15′ and 100% of contrast. (**f**,**g**) Averaged PVEPs obtained with stimulus contrast of 100% in the RP1 group with the check size of 15′ and in the RP2 group with the check size of 60′, respectively.

Amplitude and latency of the averaged PVEPs obtained in control C1 group subjects with stimuli of 78% and 100% of contrast were similar (Figure 2a,b). However, stimuli of 16% and 6% reduced the amplitude and delayed the latency of PVEPs components (Figure 2c,d). Both groups of RP patients (Figure 2f,g) showed smaller amplitudes and longer latencies of PVEPs when compared with their control C2 group (Figure 2e).The differences in P100 amplitude between the RP1 and RP2 groups were significant in all check widths. In contrast, no significant differences were observed in latency.

**Figure 3:**
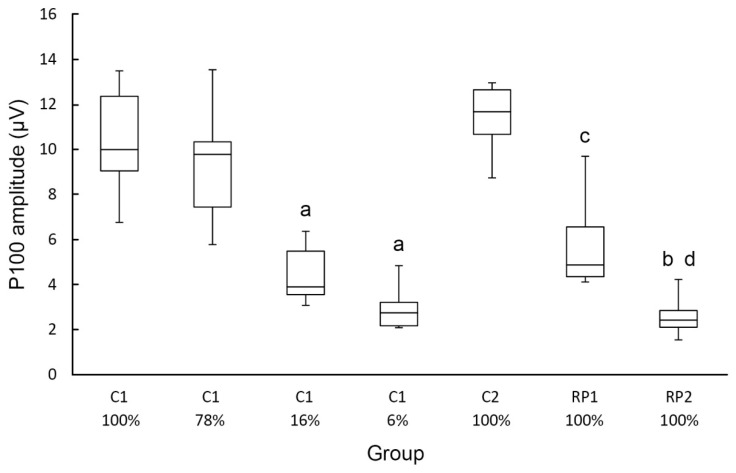
Box plots of the P100 amplitude data of the experimental groups shown in Figure 2. The contrast used in each group is indicated. a *p* < 0.001 compared with 100% of contrast of C1; b *p* < 0.001, c *p* < 0.05 compared with C2; d *p* < 0.01 compared with RP1.

Figure 3 shows a box plot where the differences in the amplitude of the P100 of the different groups investigated can be better appreciated. The figure shows the data obtained with the check widths where the largest P100 amplitude in the different experimental groups was obtained (15′ for C1 and C2, and 60′ for RP1 and RP2).

**Figure 4:**
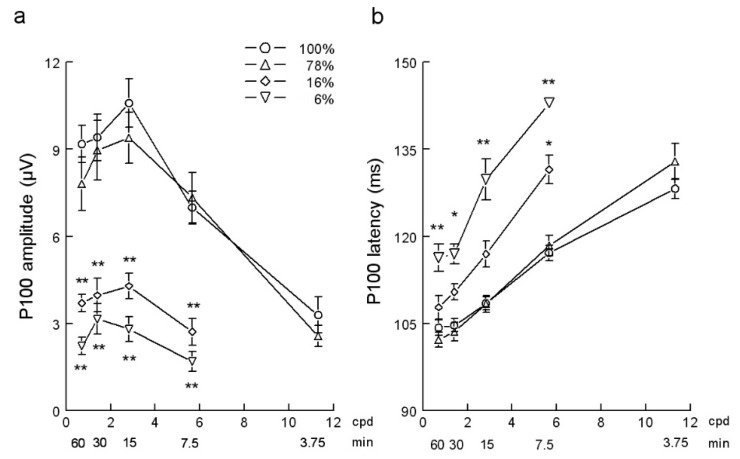
(**a**) P100 amplitude-check size functions and (**b**) P100 latency-check size functions obtained in the C1 control group with the four contrasts studied. Mean ±SEM values are depicted. * *p* < 0.05, ** *p* < 0.001 compared with the corresponding stimulus at 100% of contrast. Cpd, cycles per degree; min, minutes of arc.

**Figure 5:**
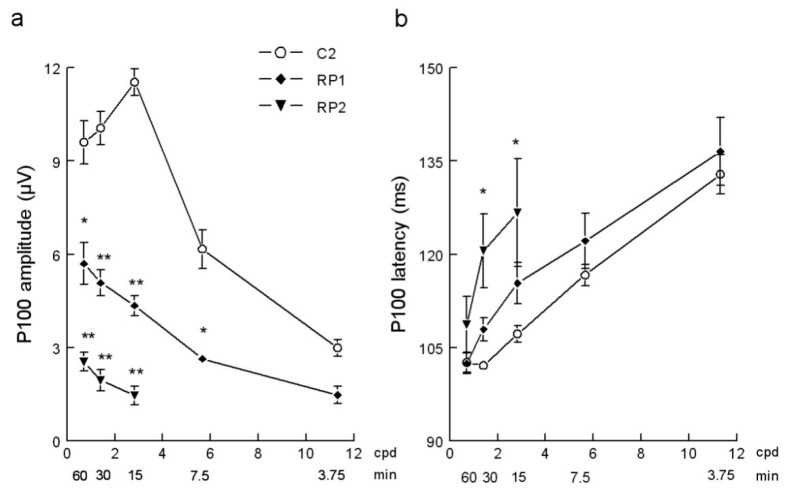
(**a**) P100 amplitude-check size functions and (**b**) P100 latency-check size functions obtained in the C2 control and in the RP1 and RP2 groups with 100% of stimulus contrast. Mean ± SEM values are depicted. * *p* < 0.05, ** *p* < 0.001 compared with the corresponding control stimulus. Cpd, cycles per degree; min, minutes of arc.

Spatial functions of the P100 VEP component obtained in the control C1 group are shown in Figure 4 and that of C2, RP1, and RP2 groups are shown in Figure 5. Control C1 group shows similar P100 amplitude-check width functions for stimuli of 100% and 78% of contrast (Figure 4a). However, smaller contrasts (16% and 6%) produced a significant (*p* < 0.001) over-all reduction of the P100 amplitude. With these two low contrasts, PVEPs were non recordable at the smallest check width tested of 3.75′. The largest amplitude of P100 was obtained with the check size of 15′ for stimuli of 100%, 78%, and 16% of contrast and with 30′ for the contrast of 6%. On the other hand, the P100 latency-check width functions of the C1 control group showed longer P100 latencies with stimuli of 16% and 6% in all check sizes tested (Figure 4b).

Compared with age equivalent controls (C2 group), the P100 amplitude-check width function obtained in RP patients showed an overall reduction of the amplitude (Figure 5a). In RP1 group, the decrease in P100 amplitude was significant at the check sizes 7.5′ and 60′ (*p* < 0.05) and at 15′ and 30′ (*p* < 0.001). In group RP2, PVEPs were only recorded with the three biggest check sizes tested and P100 amplitudes were significantly (*p* < 0.001) smaller than corresponding controls. The latency of the P100 in RP patients increased (Figure 5b) and was significant in the RP2 group of patients at the check sizes of 15′ and 30′ (*p* < 0.05).

## 4. Discussion

In this study, we described for the first time the P100 amplitude- and latency-check size functions of RP patients, obtained from PVEPs recordings. Data show that RP produces a reduction of amplitude and increment of latency of the P100 with all check sizes tested. The P100 amplitude-check size function of the patients not only differs with controls in the overall reduction of amplitude, but also in the check width at which the P100 reaches the maximum mean value. In both groups of RP patients, the peak amplitude of P100 was obtained with the check width of 60′ instead the 15′ of controls. Both functions reveal that the decrease in amplitude and increase in latency of the P100 in RP patients occurs independently of the stimuli check size, at least in the range analyzed, and that the peak of the P100 amplitude is obtained with a smaller spatial frequency (larger check size) than control normal subjects. These results show that RP alters the activity of the visual cortex in a broader manner than previously described [13,14,17].

PVEPs represent the response of visual cortex to appropriate stimulation of the ganglion cells in the central retina [12,18]. It is thought that the differential effects of check size, revealed by the P100 amplitude- and latency-spatial functions, are due to the contributions of different populations of retinal ganglion cells, according to their morphological and functional properties [19,20]. Thus, ganglion cells most sensitive to small check size stimuli have a predominantly foveal location, smaller receptive fields, and slower conduction velocity. On the other hand, the visual system is composed by two main processing streams, the parvocellular and magnocellular pathways, with different functional properties. The parvocellular system, that includes more than the 70% of the retinal ganglion cells, is responsible for visual resolution and chromatic processing, whereas the magnocellular is responsible for the detection of achromatic patterns of low spatial frequency and contrast [21,22]. The differences showed by the P100 spatial functions, obtained with high and low contrasts stimuli (see Figure 4), may reflect the differential participation of the two visual pathways. Thus, the lower P100 amplitude obtained with contrasts of 16% and 6%, together with the absence of response to the smallest check size used, indicates that P100 spatial functions obtained with low contrast are mainly due to the implication of the magnocellular system. As shown in Figure 5, the P100 spatial functions in RP patients, obtained with high contrast (100%) stimuli, were similar in several aspects to that obtained in control subjects with low contrasts (16% and 6%) stimuli. Thus, in both cases, it was found an overall reduction of P100 amplitude, peak amplitude with larger check size and non-recordable PVEPs with high spatial frequency stimuli. These results suggest contrast detection alteration in these patients. In other words, it is as if high contrast stimuli were perceived by RP patients as normal subjects perceive low contrast stimuli. Visual handicaps in RP patients are not only due to the loss of photoreceptors. The retinal dystrophy in RP, regardless of the initiating event or genetic defect, also implies profound functional and morphological changes in the retina, including glial transformations, neuronal translocations, neuronal loss, and the emergence of ectopic neurite complexes and creation of abnormal circuitries [23]. Thus, the alterations in the P100 amplitude- and latency-check size functions in RP must also be related to the complex retinal remodeling that characterizes RP retinal degeneration and that affects both the parvocellular and the magnocellular pathways [24]. The P100 is a relatively late component of PVEPs. There is evidence that P100, generated by the activity of pyramidal cells in the primary visual cortex, does not only depend on thalamic input, but also on top-down signals from the extrastriate visual areas [25]. Furthermore, the amplitude and latency of PVEPs can also be modulated by the activation of visual attention neural networks [26]. Thus, the increase in latency of P100 in RP patients may be due to deficits in synchronization and integration of subcortical and cortical inputs by the primary visual cortex induced by the altered retinal outputs. Given that RP includes various types of retinal dystrophies, it would be interesting to confirm these preliminary results in future studies with more RP patients.

## 5. Conclusions

In summary, PVEPs spatial functions reveal that RP induces significant changes in the electrophysiological activity of the visual cortex, particularly the neural processing of stimulus contrast. The impact of the RP retinal degeneration in visual cortex functions must be considered in the design of suitable vision rescue strategies in patients with RP.

## Figures and Tables

**Table 1:** Clinical characteristics of the patients.

Patient	Sex	Age	Visual	Inheritance
		(Years)	Acuity	Pattern
Group RP1				
1	Female	54	0.40	S
2	Female	51	0.67	AR
3	Male	35	1.00	AR
4	Male	25	0.67	AD
5	Female	23	1.00	AD
6	Female	50	0.40	S
7	Female	35	0.40	AD
8	Male	30	0.40	AD
Group RP2				
9	Male	52	0.40	S
10	Female	40	0.40	S
11	Female	48	0.20	AD
12	Female	29	0.10	AR
13	Female	52	0.05	AR
14	Female	57	0.40	AR
15	Female	38	0.67	S
16	Female	48	0.29	S

AD, autosomal dominant; AR, autosomal recessive; S, simplex.

**Table 2:** Amplitude and latency values (mean ± SEM) of P100 in healthy controls (C1, C2) and RP patients (R1, R2).

CheckWidth	Group																				
(cpd)	C1												C2			RP1			RP2		
	100%			78%			16%			6%			100%			100%			100%		
	Amplitude (µV)																				
0.71	9.18	±	2.05	7.81	±	2.89	3.70	±	0.97 ^a^	2.22	±	0.96 ^a^	9.60	±	2.18	5.70	±	1.93 ^d^	2.54	±	0.84 ^c,f^
1.41	9.41	±	2.53	8.97	±	3.27	3.97	±	1.84 ^a^	3.15	±	1.68 ^a^	10.06	±	1.70	5.08	±	1.15 ^c^	1.95	±	0.89 ^c,e^
2.83	10.59	±	2.66	9.39	±	2.79	4.29	±	1.36 ^a^	2.80	±	1.14 ^a^	11.54	±	1.37	4.35	±	0.90 ^c^	1.46	±	0.59 ^c,e^
5.66	6.99	±	1.81	7.33	±	2.72	2.71	±	1.31 ^a^	1.69	±	0.60 ^a^	6.17	±	1.97	2.64	±	0.45 ^d^			
11.31	3.29	±	1.85	2.56	±	1.09							2.99	±	0.81	1.47	±	0.57			
	Latency (ms)																				
0.71	104.27	±	4.40	102.22	±	4.05	107.79	±	6.45	116.31	±	7.57 ^a^	102.61	±	4.91	102.44	±	4.60	108.71	±	12.69
1.41	104.66	±	3.52	103.58	±	5.17	110.39	±	4.43	116.95	±	5.56 ^b^	102.12	±	2.62	107.93	±	5.29	120.57	±	15.68 ^d^
2.83	108.45	±	3.46	108.37	±	4.61	116.93	±	7.04	129.77	±	9.40 ^a^	107.19	±	4.19	115.36	±	9.50	126.70	±	17.38 ^d^
5.66	117.13	±	4.14	118.41	±	5.65	131.49	±	6.96 ^b^	143.00	±	1.47 ^a^	116.66	±	5.37	122.15	±	10.82			
11.31	128.22	±	5.11	132.89	±	9.33							132.86	±	9.37	136.53	±	10.97			

^a^*p* < 0.001, ^b^ *p* < 0.05 compared with 100% of contrast of C1; ^c^ *p* < 0.001, ^d^ *p* < 0.05 compared with C2; ^e^ *p* < 0.001, ^f^ *p* < 0.01 compared with RP1; cpd, cycles per degree.
